# Toxicological Assessment of Oligofructans Derived from Raw Sugar Fermentation by *Bacillus subtilis* TISTR 001 and Their Modulatory Effects on Rat Gut Microbiota

**DOI:** 10.3390/nu18132191

**Published:** 2026-07-05

**Authors:** Sirinya Taya, Boontiwa Ninchan, Sonthaya Umsumarng, Jutamat Klinsoda, Rawiwan Wongpoomchai, Charatda Punvittayagul

**Affiliations:** 1 Multidisciplinary Research Institute, Chiang Mai University, Chiang Mai 50200, Thailand; sirinya.t@cmu.ac.th; 2 Department of Forensic Medicine, Faculty of Medicine, Chiang Mai University, Chiang Mai 50200, Thailand; 3 Department of Biotechnology, Faculty of Agro-Industry, Kasetsart University, Bangkok 10900, Thailand; boontiwa.ni@ku.th; 4 Faculty of Veterinary Medicine, Chiang Mai University, Chiang Mai 50100, Thailand; sonthaya.u@cmu.ac.th; 5 Institute of Food Research and Product Development, Kasetsart University, Bangkok 10900, Thailand; ifrjmk@ku.ac.th; 6 Department of Biochemistry, Faculty of Medicine, Chiang Mai University, Chiang Mai 50200, Thailand; rawiwan.wong@cmu.ac.th

**Keywords:** oligofructans, *Bacillus subtilis*, raw sugar, acute toxicity, subchronic toxicity, gut microbiota

## Abstract

Background/Objectives: Oligofructans are a category of non-digestible carbohydrates with beneficial effects on gut health and microbiota modulation. In this study, oligofructans were produced from raw sugar using *Bacillus subtilis* TISTR 001, and their safety and effects on the gut microbiota were assessed in rats. Methods: The acute toxicity assessment consisted of administering a single oral dose of 2000 mg/kg body weight (bw), whereas the subchronic toxicity assessment included oral dosages of 200, 600, and 2000 mg/kg/day for 90 days. Results: In the acute toxicity test, no mortality or toxicity was observed in the rats treated with a single dose of oligofructans during the 14-day observation period. The median lethal dose (LD_50_) of the oligofructans was >2000 mg/kg bw. In the subchronic toxicity study, daily oligofructans doses of 200, 600, and 2000 mg/kg bw for 90 days did not cause lethality or toxic clinical symptoms in rats of either sex. Furthermore, no treatment-related adverse effects of oligofructans on the hematological and biochemical parameters or organ histopathology were observed in the treatment and satellite groups. Hence, the no-observed-adverse-effect level (NOAEL) of oligofructans under the study’s test conditions was confirmed as 2000 mg/kg/day. Conclusion: No adverse effects were observed in either acute or subchronic toxicity studies at doses up to 2000 mg/kg/day. Moreover, oligofructans modulated the gut microbiota by promoting the growth of potentially beneficial commensal bacteria and reducing the taxa associated with inflammation or metabolic dysfunction. However, further studies are required to confirm these microbiome-related changes in humans.

## 1. Introduction

Oligofructans are carbohydrates composed of fructooligosaccharides (FOS) and fructans. FOSs are short-chain oligosaccharides consisting of repeating fructose units with a degree of polymerization (DP) ≤ 10, including compounds such as kestose, nystose, and 1-fructofuranosyl-D-nystose. In contrast, fructans are longer-chain fructose polymers with a DP > 10, such as inulin and levan. Structurally, oligofructans consist of fructose units linked via β-(2→1) or β-(2→6) glycosidic bonds, typically with a terminal α-glycosidic linkage to a glucose residue [1,2,3]. These compounds are widely used in the food, pharmaceutical, and nutraceutical industries owing to their unique properties and health benefits [4,5,6,7,8,9]. In particular, oligofructans exhibit several health benefits, such as colon health benefits, immune system modulation, and anti-inflammatory and antitumor properties [7,9,10,11]. Despite their broad applications, large-scale industrial production remains challenging [12]. Therefore, developing innovative and cost-effective approaches to improve yield and productivity is important for their commercial production.

Ninchan and Noidee first demonstrated that *Bacillus subtilis* TISTR 001 can convert sugarcane juice into functional oligofructans with prebiotic activity by promoting the growth of *Bifidobacterium bifidum* TISTR 2129 and suppressing pathogens, including *Escherichia coli* TISTR 073 and *Salmonella enterica serovar Enteritidis* S003 [2]. Building on this, they optimized production by first improving the fermentation medium (20 °Brix sugarcane juice plus meat extract, peptone, ammonium sulfate, and magnesium sulfate at pH 6.8), achieving 3.79% (*w*/*v*) oligofructans after 84 h [3]. A subsequent statistical optimization (using a Box–Behnken experimental design) identified optimal conditions of 35 °Brix substrate, pH 6.5, 32.5 °C, and 250 rpm for 48 h, with a predicted yield of 88.64 g/L; sucrose produced the highest observed yield (75.86 g/L), while raw sugar and sugarcane juice were viable alternatives, and molasses performed poorly due to inhibitory impurities [1]. In addition to process optimization and production efficiency, the beneficial effects of oligofructans have been investigated. Punvittayagul et al. studied the anti-carcinogenic properties of oligofructans derived from raw sugar fermented using *B*. *subtilis* TISTR 001 in a 1,2-dimethylhydrazine (DMH)-induced rat model of colon carcinogenesis [13]. Oligofructans did not promote colonic aberrant crypt foci (ACF) formation, and oligofructans administration markedly decreased the number of ACF in DMH-treated rats. Furthermore, the preventive mechanisms may have involved the modulation of genes associated with inflammation, cell growth, and apoptosis. Given prior observations of anti-carcinogenic effects, it is essential to establish a comprehensive safety profile before potential human use.

The use of oligofructans produced from raw sugar fermentation using *B*. *subtilis* TISTR 001 in the food, pharmaceutical, and nutraceutical industries requires extensive safety assessments. Any novel ingredient intended for human consumption must be thoroughly toxicologically assessed to ensure public health protection and compliance with strict regulatory frameworks established by organizations, such as the United States Food and Drug Administration, European Food Safety Authority, and national food safety boards [14,15,16]. Acute toxicity assessments are crucial for identifying possible adverse effects of a single dose of a chemical. These data are used to categorize compounds into toxicity classifications. In particular, acute toxicity endpoints such as mortality and clinical symptoms are utilized to estimate the LD_50_, and the obtained values are used to designate hazard categories [17,18]. Subchronic toxicity studies, which typically span 28–90 days, aim to assess the consequences of repeated exposure to a chemical and determine potential target organ toxicity or cumulative effects. These studies are essential for hazard characterization and risk assessment in various fields, including pharmaceuticals, industrial chemicals, and food safety [19,20]. These evaluations are carefully designed in accordance with internationally recognized procedures, such as those established by the Organization for Economic Cooperation and Development (OECD), which ensure transparency, reproducibility, and a scientific foundation for risk assessment. Information on the toxicological safety of oligofructans produced by *B*. *subtilis* TISTR 001 under an optimized raw sugar fermentation process remains limited, particularly regarding acute and subchronic toxicity tests, as well as the microbiota-modulating effects in vivo. The present study addresses these gaps by evaluating both the safety and gut microbiota-modulating effects of oligofructans derived from raw sugar fermentation by *B*. *subtilis* TISTR 001 in rats.

## 2. Materials and Methods

### 2.1. Chemicals

The culture media, including nutrient broth, yeast extract, meat extract, and peptone, were purchased from HiMedia Laboratories Pvt. Ltd. (Thane, India). Ammonium sulfate and magnesium sulfate heptahydrate were purchased from Ajax Finechem (Sydney, Australia). The standard sugars, sucrose, glucose, and fructose, and standard short-chain fructooligosaccharides, 1-kestose, nystose, and 1-fructofuranosyl-D-nystose, were supplied by Sigma Aldrich (St. Louis, MO, USA). All chemicals were of analytical grade.

### 2.2. Preparation of B. subtilis TISTR 001 Starter Culture

*Bacillus subtilis* TISTR 001 was provided by the Thailand Institute of Scientific and Technological Research (Technopolis Science Park, Thailand). The starter culture was established by inoculating flasks containing 400 mL of nutrient broth (NB) with *B*. *subtilis* TISTR 001 colonies and incubating at 37 °C with a shaking speed of 200 rpm for 24 h. The culture was subsequently transferred to 3.6 L of NB liquid medium supplemented with a 0.1% (*w*/*v*) sucrose solution. Incubation was conducted for 10 h to facilitate the transfer of the starting cultures into 50 L fermenters. All the procedures were performed using aseptic techniques.

### 2.3. Oligofructans Production

Oligofructans production from raw sugar fermentation by *B*. *subtilis* TISTR 001 was performed as previously described by Noidee and Ninchan [1]. The raw sugar was produced and supplied by Buriram Sugar Factory Co., Ltd. (Buriram, Thailand). A 35% (*w*/*v*) raw sugar solution was prepared and added to yeast extract (2 g/L), meat extract (3 g/L), peptone (5 g/L), ammonium sulfate ((NH_4_)_2_SO_4_, 2 g/L), and magnesium sulfate heptahydrate (MgSO_4_·7H_2_O, 0.6 g/L). Thereafter, the pH was adjusted to 6.8 using 0.1 N sodium hydroxide (KemAus^TM^, Castle Hill, Australia) before being used as the culture medium. Sterile culture medium (36 L) was prepared in a 50 L fermenter. Subsequently, 4 L of the prepared *B*. *subtilis* starter culture was transferred into the sterilized medium and fermented at 30 °C, with a shaking speed of 150 rpm and aeration of 0.33 vvm for 48 h. *Bacillus subtilis* cells were then isolated by continuous centrifugation. Oligofructans were precipitated by mixing the fermented solution with cold absolute ethanol (99.8% purity, food grade) at a ratio of 1:2 (*v*/*v*), followed by incubation at 4 °C in a refrigerator for 48 h. A single precipitation step was performed for oligofructans recovery. The oligofructans were dried using a spray dryer (Spray dryer model B-290, Buchi, Tokyo, Japan) with maltodextrin at a concentration of 1% (*w*/*v*). The spray-drying process was performed at an inlet temperature of 140 °C and an outlet temperature of 85 °C, with a feed flow rate of 1.4–6.2 mL/min. The oligofructans composition was investigated, including the amount of reducing sugar, total oligofructans content in the form of free fructose, and amounts of sucrose, glucose, fructose, and short-chain FOSs. All composition analyses were performed in triplicate.

### 2.4. Analysis of Oligofructans Composition

#### 2.4.1. Determination of Reducing Sugar Content Using the Somogyi-Nelson Method

This method was followed by Noidee and Ninchan [1]. The oligofructans solution was mixed with 1 mL of alkaline copper reagent, shaken well and heated in hot water for 15 min, and then rapidly cooled in an ice bath. After the addition of 1 mL of Nelson’s reagent, the mixture was incubated in the dark at room temperature for 30 min. Then, 5 mL of distilled water was added and mixed thoroughly. The optical density of the mixture was measured at 520 nm using a fructose standard to quantify the reducing sugars.

#### 2.4.2. Analysis of Total Oligofructans Content in the Form of Free Fructose

Total oligofructans were analyzed based on Goncalves et al. [21] and Ninchan and Noidee [2], with slight modifications. A 10% (*w*/*v*) oligofructans solution was prepared, and 1 mL of 10% oligofructans solution was mixed with 2 mL of 0.1 N hydrochloric acid and boiled for 30 min to hydrolyze the oligosaccharide and fructan polymers into free fructose. The reducing sugar content was assessed using the Somogyi-Nelson method and quantified using a fructose standard curve. The quantities of FOSs and total fructans were determined based on total free fructose content.

#### 2.4.3. Determination of Glucose, Fructose, Sucrose, and Short-Chain FOS Content

Sugar content, including glucose, fructose, sucrose, and short-chain FOSs, was analyzed as described by Ninchan and Noidee [2]. Determination of the sugar contents in oligofructans was performed using high-performance liquid chromatography (Shimadzu Corporation, Kyoto, Japan), with a refractive index detector (RID-10A, Shimadzu Corporation) and a Shodex Asahipak NH2P-50 4E (4.6 mm I.D. × 250 mm, Showa Denko America, Inc., New York, NY, USA) at 40 °C. Deionized water and 99.9% acetonitrile (ratio 25:75, *v*/*v*) were used as the mobile phase at a flow rate of 1 mL/min, with standard sugars (sucrose, glucose, and fructose) and FOS standards (1-kestose, nystose, and 1-fructofuranosyl-D-nystose).

### 2.5. Animals

Male and female Sprague-Dawley rats were purchased from Nomura Siam International Co., Ltd. (Bangkok, Thailand). In the acute oral toxicity study, 10 female rats were used, while in the 90-day repeated oral toxicity study, 50 male and 50 female rats were used. The rats were acclimatized for one week prior to starting the experiments. The animals were housed under standard environmental conditions, maintained on a 12 h dark–light cycle, and given free access to drinking water and a commercial pelleted diet (SmartHeart Hamster Food Complete & Balanced, product code 8HT41; Perfect Companion Group Co., Ltd., Samutprakarn, Thailand). Animals were fed a commercial pelleted food. Ground corn, soybean meal, full-fat soybeans, fish meal, poultry by-product meal, vitamins and minerals, and antioxidants are listed as ingredients by the manufacturer. Guaranteed analysis: Crude protein > 24.0%, crude fat > 4.5%, crude fiber < 5.0%, moisture < 10.0%, ash < 10.0%, metabolizable energy 314 kcal per 100 g. The animal study protocol for acute toxicity was approved by the Institutional Animal Care and Use Committee of the Faculty of Medicine, Chiang Mai University (Protocol No. 29/2566; approved on 28 August 2023). Moreover, the Animal Care and Use Committee of Chiang Mai University approved the animal study protocol for subchronic toxicity (Protocol No. 2567/RT-0004, approved on 5 January 2024) and the use of preserved tissues in the project (Protocol No. 2567/RT-0004. C1, approved on 14 February 2024). All procedures were conducted in accordance with relevant guidelines and regulations, and all studies complied with the Animal Research: Reporting of In Vivo Experiments guidelines.

### 2.6. Acute Toxicity Study

An acute toxicity study of oligofructans was conducted in accordance with OECD Guideline 420 [17] using eight-week-old female Sprague-Dawley rats. Animals were body-weight-stratified and randomly assigned to two groups of five rats each. One rat from each group was fasted for 16 h prior to administration and then received a single oligofructans dose of 2000 mg/kg body weight (bw) or vehicle control (distilled water) by oral gavage. The animals were observed for clinical signs of toxicity, including changes in fur, depressed activity, vomiting, convulsions, muscle spasms, tremors, weakness or fatigue, and watery diarrhea, and mortality during the first 6 h after drug administration and again at 24 h. If no mortality occurred, the remaining animals were administered the drug until all five animals in each group were treated. Thereafter, all the animals were monitored once daily for 14 days for signs of toxicity and mortality. The body weights of the rats were measured daily, and food and water intake were recorded once a week. All rats were euthanized by an overdose of isoflurane at the end of the experiment. The major internal organs, including lung, heart, thymus, stomach, liver, kidney, adrenal gland, spleen, pancreas, and ovary, were then collected and weighed, and a gross examination was performed.

### 2.7. Subchronic Toxicity Study

The subchronic toxicity study of oligofructans complied with OECD guideline no. 408 [20]. The high dose of oligofructans (2000 mg/kg, bw) was selected based on the acute toxicity test. The low and medium doses were set at 200 and 600 mg/kg bw, respectively, with dose levels spaced approximately threefold apart. Forty male and forty female Sprague-Dawley rats (six weeks old) were body-weight-stratified and randomly assigned to four groups (10 females and 10 males per group). Rats received either vehicle (distilled water) or different oligofructans dosages (200, 600, and 2000 mg/kg bw once daily) via oral gavage for 90 days. Additionally, ten male and ten female Sprague-Dawley rats were body-weight–stratified and randomly assigned to two groups (five females and five males per group): satellite control and satellite oligofructans groups. Rats were orally administered vehicle and a high oligofructans dose (2000 mg/kg bw) for 90 days, followed by a subsequent 28-day period for potential reversibility or persistence of any toxic effects. The animals were regularly observed for mortality and general clinical signs, including changes in appearance and behavior, such as coat condition, posture, locomotion, lethargy, tremors, and diarrhea. The body weight, diet, and water consumption were recorded weekly throughout the experiment. At the end of the experiment, all rats were fasted overnight and anesthetized via high-dose isoflurane inhalation. Rat feces were collected from the anus and stored at −80 °C for analysis of gut microbiota. Blood samples were collected to determine hematological and clinical biochemical parameters. The organs were removed for organ weight measurement and histopathological examination.

### 2.8. Hematology and Clinical Biochemistry

Hematological and clinical biochemical measurements were performed using an automated hematology analyzer Mindray BC-5300 Vet (Shenzhen Mindray Bio-Medical Electronics Co., Ltd., Shenzhen, China) and an automated BX-3010 chemistry analyzer (Sysmex Asia Pacific Pte Ltd., Singapore) in a hematology laboratory at the Northern Regional Veterinary Healthcare and Learning Center, Faculty of Veterinary Medicine, Chiang Mai University and Veterinary Diagnostic Center Co., Ltd., Chiang Mai, Thailand.

The following hematological parameters were determined: red blood cell (RBC) count, hemoglobin (Hb), hematocrit (Hct), mean corpuscular volume (MCV), mean corpuscular hemoglobin (MCH), MCH concentration (MCHC), red blood cell distribution width (RDW), white blood cell (WBC) count, neutrophils (Neu), lymphocytes (Lym), monocytes (Mono), eosinophils (Eos), basophils (Baso), and platelet count (PLT).

The following biochemical parameters were determined: glucose, blood urea nitrogen (BUN), creatinine, aspartate aminotransferase (AST), alanine aminotransferase (ALT), alkaline phosphatase (ALP), total protein, albumin, globulin, total bilirubin, direct bilirubin, cholesterol, triglycerides, high-density lipoprotein-cholesterol (HDL-C), low-density lipoprotein-cholesterol (LDL-C), and uric acid. Moreover, serum electrolytes, including sodium (Na^+^), potassium (K^+^), chloride (Cl^−^), and total carbon dioxide concentration (TCO_2_), were measured.

### 2.9. Histopathology

Internal and reproductive organs, including the brain, lung, heart, thymus, stomach, liver, kidney, adrenal gland, spleen, pancreas, prostate, epididymis, seminal vesicle, testis, and ovary, were collected for histopathological examination. After weight measurement, the organs were fixed in 10% buffered formalin. Fixed tissues were embedded in paraffin, sectioned, and stained with hematoxylin and eosin (H&E) in accordance with the conventional histological procedures described by Suvarna et al. [22]. Histopathology was conducted in accordance with OECD guideline no. 408. The histopathological examination was performed on preserved organs from all animals in the control and high-dose groups. One representative section per organ per animal was examined, and all animals in these groups were also evaluated. Tissues from all other dosage groups were examined only if treatment-related changes were identified in the high-dose group. Histopathological evaluation was based on qualitative assessment of lesions using an optical microscope (ZEISS Axio Observer 7, Langen, Germany), and photomicrographs were captured. Histopathological evaluation was performed in a blinded manner, with the pathologist blinded to the treatment group allocation.

### 2.10. Analysis of Fecal Gut Microbiota

The gut microbiota in the feces of male and female rats (*n* = 5 per sex per group) in the control and 2000 mg/kg bw oligofructans-treated groups was analyzed. The high dose was selected because it represents the maximum administered dose used for safety evaluation and is most relevant for toxicological interpretation. DNA was extracted from rat feces using a TIANamp Stool DNA Kit (Tiangen Biotech, Beijing, China) following the manufacturer’s instructions. The concentration and purity of DNA were evaluated using a NanoDrop One spectrophotometer (Thermo Scientific, Waltham, MA, USA). The DNA samples were submitted to a sequencing company (Novogene AIT Genomics, Singapore). The V3–V4 region of the bacterial 16S rRNA gene was amplified using primers 341F (5′-CCTAYGGGRBGCASCAG-3′) and 806R (5′-GGACTACNNGGGTATCTAAT-3′). The amplicons were sequenced using an Illumina NovaSeq 6000 (Illumina, San Diego, CA, USA).

Sequences were processed using QIIME2 software (version 202202). Paired-end reads were demultiplexed based on their unique barcodes and truncated by removing the barcodes and primer sequences. The paired-end reads were merged, and quality filtering of the raw tags was performed using Fastp (version 0.23.1) to obtain high-quality clean tags. Quality filtering was performed to remove reads containing ambiguous bases and low-quality sequences. Chimeric sequences were subsequently identified and removed by comparison against the SILVA database using vSearch (version 2.16.0). The resulting high-quality non-chimeric sequences were denoised using the DADA2 plugin in QIIME2 to generate Amplicon Sequence Variants (ASVs). Taxonomic annotation of each ASV was performed using the classify-sklearn algorithm against the SILVA 138.2 database. Finally, taxonomic abundance tables were constructed at the genus level by integrating the ASV annotations with the feature tables of each sample. Multiple sequence alignment was performed to align homologous nucleotide positions among representative ASV sequences prior to phylogenetic tree construction. The resulting phylogenetic tree was subsequently used for phylogeny-based diversity analyses, including weighted and unweighted UniFrac distance calculations. To ensure comparability between samples, ASV counts were rarefied to an even sequencing depth of 96,040 reads per sample, corresponding to the sample with the lowest sequencing depth. Rarefaction curve analysis indicated adequate sequencing coverage. The rarefied dataset was used for all subsequent alpha and beta diversity analyses. To visualize the microbial community composition, the 10 most abundant taxa at the genus level were identified for each sample.

Alpha diversity was assessed using the Chao1, Shannon, and Simpson indices to evaluate microbial richness and evenness within the samples. To assess beta diversity, a principal coordinate analysis (PCoA) plot was constructed based on Bray–Curtis dissimilarity. To identify specific microbial taxa that were significantly altered by oligofructans intervention, differential abundance analysis was performed using the metagenomeSeq package in R.

### 2.11. Statistical Analysis

The data from the animal model are expressed as mean ± standard error of the mean (SEM). All analyses were performed using Prism version 8.0 software (Boston, MA, USA). For the acute toxicity test and the satellite control and satellite oligofructans-treated groups in the subchronic toxicity study, an independent *t*-test was performed to test for differences in the parametric data. For the subchronic toxicity test, the Kolmogorov–Smirnov test was used to test for normality of distribution, and the Brown-Forsythe test was used for homogeneity of variance. For normally distributed data, a comparison of the results between groups was performed using one-way analysis of variance, followed by Tukey’s multiple comparison tests. Statistical significance was set at *p* < 0.05.

For microbiota data, alpha diversity indices were analyzed using the Kruskal–Wallis test, and statistical significance was set at *p* < 0.05. Differential abundance analysis was performed using metagenomeSeq with cumulative sum scaling (CSS) normalization and a zero-inflated Gaussian (ZIG) model. Taxa with a Benjamini–Hochberg adjusted *p*-value (FDR) < 0.05 were considered significantly different between groups.

## 3. Results

### 3.1. Oligofructans Obtained from the Fermentation of Raw Sugar by B. subtilis TISTR 001

Oligofructans derived from raw sugar fermentation by *B. subtilis* TISTR 001 comprised both short-chain FOSs and fructans; however, short-chain FOSs (1-kestose and nystose) were present at low levels. In contrast, the amount of total fructose polymers was approximately 71.24 ± 0.13% (*w*/*w*) and was assessed based on the amount of free fructose sugars associated with the chains of FOSs and fructans or oligofructans. Table 1 presents the results of the study. Oligofructans may contain glucose and fructose sugars as a result of the heat generated during spray drying. Moreover, sucrose is completely depleted during fermentation, thereby yielding oligofructose. A purity of 92.11 ± 0.72% (*w*/*w*) relative to the total polymer determined as free fructose and a degree of polymerization of 3–30 for oligofructans have previously been reported [13].

### 3.2. Acute Toxicity Test of Oligofructans

Oligofructans administered at a dose of 2000 mg/kg bw had no adverse effects on rat behavior over 14 days. Furthermore, no mortality was detected at the administered dose, and no adverse effects on rat weight were observed. Gross examination of various organs revealed no abnormalities, and the median oligofructans lethal dose (LD_50_) was estimated to be >2000 mg/kg bw.

### 3.3. Subchronic Toxicity Test of Oligofructans

#### 3.3.1. Body Weight and Consumption of Diet and Water

Throughout the study period, all animals exhibited normal growth and higher body weights than their initial weights. In female rats, oligofructans doses of 200, 600, and 2000 mg/kg bw did not substantially affect the final body weights in the treatment groups compared with those in the control group. Additionally, an oligofructans dose of 2000 mg/kg bw did not cause a pronounced difference in final body weight in the satellite group compared with that in the satellite control group (Table 2). In male rats, no notable differences in final body weights were observed among the treatment groups. However, a pronounced reduction in final body weight was found in the satellite group receiving 2000 mg/kg bw of oligofructans compared with that in the satellite control group (Table 3).

In female rats, diet consumption in the oligofructans-treated groups showed no considerable differences compared with that of the control group. In contrast, female rats in the satellite group administered 2000 mg/kg bw of oligofructans exhibited a marked decrease in diet consumption compared with that in the satellite control group (Table 2). In male rats, a substantial reduction in diet consumption was observed in rats receiving the highest oligofructans dose (2000 mg/kg bw) in both the treatment and satellite groups compared with that in their corresponding control groups (Table 3). No noteworthy differences in water consumption were observed between the treatment and the satellite groups of female rats (Table 2). In male rats, no considerable differences in water consumption were observed between the treatment groups. However, male rats in the satellite group receiving 2000 mg/kg bw of oligofructans showed markedly lower water consumption than that in the satellite control group (Table 3).

#### 3.3.2. Organ Weight

The absolute and relative organ weights of female rats are presented in Table 4. No pronounced differences in absolute organ weights were observed between the treatment and control groups, except for a pronounced decrease in the absolute spleen weight of rats treated with 200 mg/kg bw of oligofructans. In the satellite group, no substantial differences in absolute organ weights were observed between the satellite group administered 2000 mg/kg bw of oligofructans and the satellite control group. Moreover, no substantial differences in relative organ weights were found among the treatment groups at any dose level. However, in the satellite group, female rats administered 2000 mg/kg bw of oligofructans exhibited substantially increased relative organ weights of the kidney and heart compared with those in the satellite control group.

The absolute and relative organ weights of male rats are presented in Table 5. No pronounced difference in absolute organ weight was observed between the treatment and control groups, with the exception of a pronounced increase in the absolute lung weight of rats administered 2000 mg/kg bw of oligofructans. Moreover, no substantial differences in the absolute organ weights were observed in the male satellite group. No pronounced differences in relative organ weights were detected among the treatment groups at any dosage level. Furthermore, male rats administered 2000 mg/kg bw of oligofructans exhibited a substantial increase in the relative organ weight of the lungs compared with that of the control group. A notable reduction in the relative weight of the pancreas was observed in the 200 and 600 mg/kg bw oligofructans-treated groups; however, this decrease was not observed in the highest-dose treatment group (2000 mg/kg bw). In the satellite group, the relative weight of the thymus was substantially increased in the 2000 mg/kg bw oligofructans-treated group compared with that in the corresponding control, with no substantial changes detected in other organs.

#### 3.3.3. Hematology and Serum Biochemistry Assessment

The hematological parameters of female rats are presented in Table 6. In the treatment groups, female rats administered the highest oligofructans dose (2000 mg/kg bw) exhibited a notable increase in RBC counts and Hct levels compared with those in the control group. In contrast, the WBC count and percentage of Baso in the 2000 mg/kg bw oligofructans-treated group were substantially lower than those in the control group. No other pronounced differences were observed in the remaining parameters between the treatment groups. In the satellite group, the majority of hematological parameters remained comparable, with the exception of PLT counts, which demonstrated a marked reduction in the 2000 mg/kg bw oligofructans-treated group compared with that in the satellite control group.

The hematological parameters of the male rats are summarized in Table 7. In the treatment groups, the administration of 200, 600, and 2000 mg/kg bw of oligofructans did not result in a pronounced difference in hematological parameters compared with those in the control group. Similarly, most parameters in the satellite group showed no notable differences compared with those in the satellite control group. However, a pronounced increase in the percentage of Neu was observed in rats receiving 2000 mg/kg bw of oligofructans compared with that in the satellite control group.

The biochemical and electrolyte parameters of female rats are presented in Table 8. In the treatment groups, creatinine levels were markedly increased in rats treated with 200 mg/kg bw of oligofructans, and uric acid was substantially increased in rats treated with the highest oligofructans dose compared with that in the control group. A pronounced decrease in Na^+^ and increase in K^+^ were observed in rats treated with 600 mg/kg bw of oligofructans. In the satellite group, the only statistically significant alteration was an increase in glucose levels in rats administered 2000 mg/kg bw of oligofructans compared with that in the satellite control group.

The biochemical and electrolyte parameters of male rats are summarized in Table 9. In the treatment groups, total bilirubin exhibited a pronounced decrease in rats treated with 200 mg/kg bw of oligofructans compared with that in the control group. Rats in the highest dose group of oligofructans (2000 mg/kg bw) showed a substantial reduction in creatinine levels and a pronounced increase in TCO_2_ levels. In the satellite group, treatment with 2000 mg/kg bw of oligofructans substantially increased globulin levels compared with those in the corresponding control. No other notable differences were observed between the remaining parameters in the satellite groups.

#### 3.3.4. Histopathological Assessment

**Figure 1:**
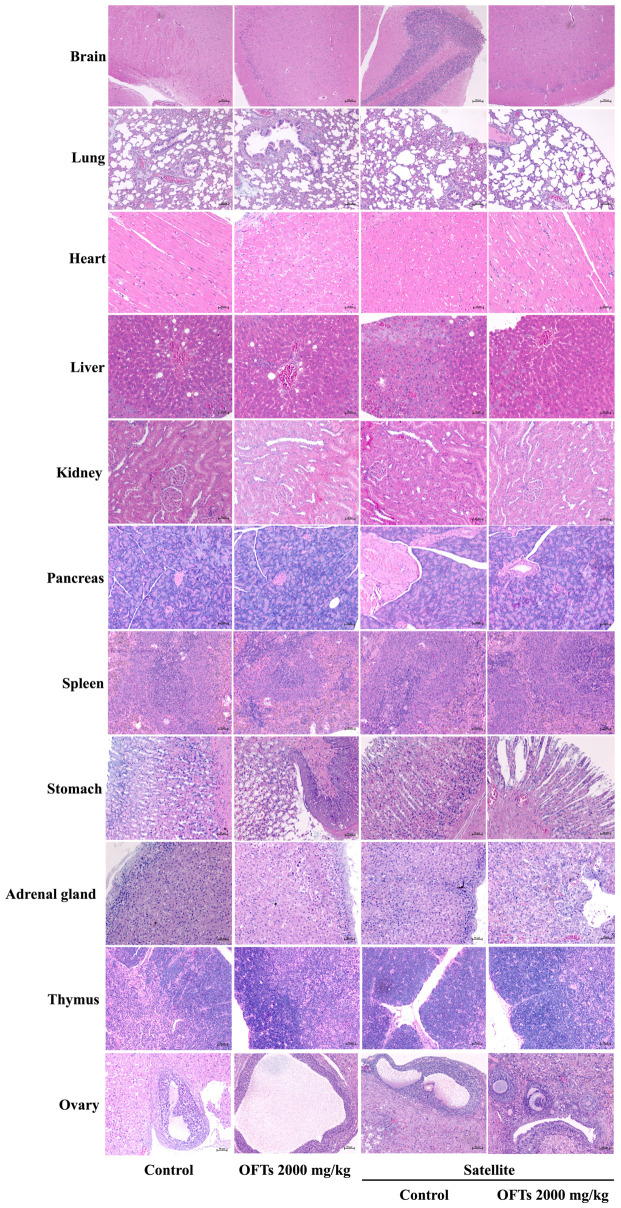
Representative photomicrographs of histopathological sections from female rats following the subchronic oral administration of oligofructans (OFTs). The columns (from left to right) correspond to the normal control, OFTs 2000 mg/kg bw, satellite control, and satellite OFTs 2000 mg/kg bw groups. Tissues were stained with hematoxylin and eosin (H&E). The brain and lung were examined at 10× magnification, whereas the heart, liver, kidney, pancreas, spleen, stomach, adrenal gland, thymus, and the ovary (female reproductive organ) were examined at 20× magnification. Scale bars: Brain and lung, 100 μm; all other tissues, 50 μm. *n* = 10 per group (treatment groups), *n* = 5 per group (satellite groups).

**Figure 2:**
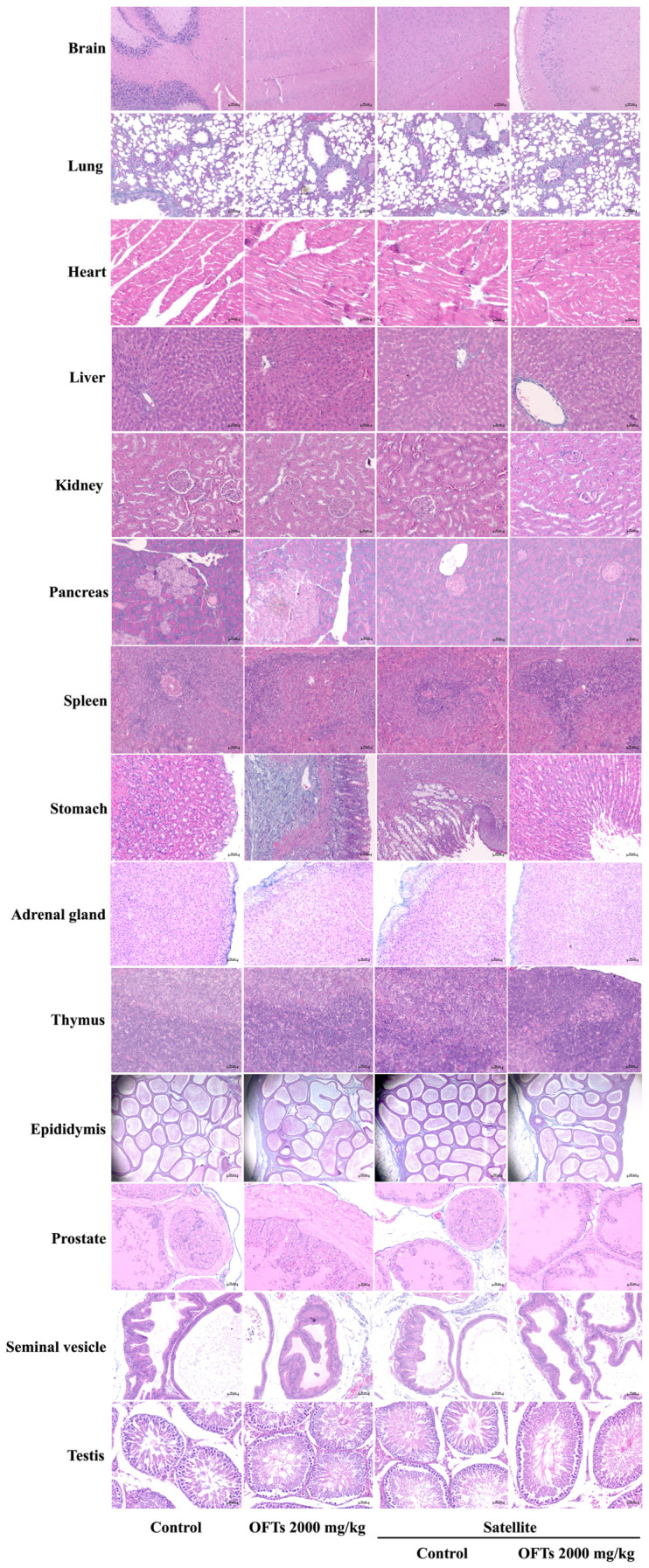
Representative photomicrographs of histopathological sections from male rats following the subchronic oral administration of oligofructans (OFTs). The columns (from left to right) correspond to the normal control, OFTs 2000 mg/kg bw, satellite control, and satellite OFTs 2000 mg/kg bw groups. Tissues were stained with hematoxylin and eosin (H&E). The brain, lung, and epididymis were examined at 10× magnification, whereas the heart, liver, kidney, pancreas, spleen, stomach, adrenal gland, thymus, and the remaining male reproductive organs (prostate, seminal vesicle, and testis) were examined at 20× magnification. Scale bars: Brain and lung, 100 μm; epididymis, 200 μm; all other tissues, 50 μm. *n* = 10 per group (treatment groups), *n* = 5 per group (satellite groups).

Histopathological tissue sections of various internal organs, including the brain, lung, heart, liver, kidney, pancreas, spleen, stomach, adrenal gland, thymus, female reproductive organs (ovary), and male reproductive organs (epididymis, prostate, seminal vesicle, and testis), were examined (Figure 1 and Figure 2). The histological architecture of the rats treated with 2000 mg/kg bw of oligofructans was normal throughout all examinations, with no considerable pathological lesions or cellular abnormalities. Moreover, the histological architecture was very similar to the structural integrity of the normal control and satellite groups.

Detailed microscopic analysis confirmed the absence of toxic effects on all defined tissue structures. Neither spongiosis nor demyelinated lesions were observed in the cerebral hemispheres or the cerebellum of the central nervous system. Pulmonary evaluation revealed normal tissue architecture with no inflammatory epithelial modifications in the major and small airways (bronchi, bronchioles, alveolar ducts, and alveolar sacs) or lung capillaries. Cardiac assessment revealed intact cardiomyocytes and well-preserved connective tissue networks. In the liver, the hepatocytes, hepatic sinusoids, and central veins showed normal morphology. Similarly, renal assessment revealed no structural abnormalities in the glomeruli, glomerular capillaries, or renal tubules. The pancreatic architecture was normal, and no aberrant lesions were observed in either the exocrine acinar or endocrine islet cells.

No adverse effects were observed in the lymphatic, digestive, and endocrine systems. The spleen had complete capsules with well-defined white and crimson pulps. No unusual lesions were observed in the gastric pits, glands, muscularis mucosa, or submucosa of the gastric tissues. The adrenal glands showed a normal architecture of the cortex (zona glomerulosa, fasciculata, and reticularis) and medulla. Thymic examination revealed normal cortical and medullary zones with no cellular abnormalities. In particular, the lymphocytes and epithelial cells within the thymus appeared healthy, with no signs of tumors, thymoma-like lesions, inflammation, or atrophy.

Finally, oligofructans administration had no effects on the microscopic structures of any of the evaluated male and female reproductive organs, with no evidence of pathological modification compared with that in the control and satellite groups. Rat ovaries often showed no abnormalities in female reproductive organ architecture, thereby indicating healthy folliculogenesis and normal reproductive function. Similarly, the epididymis architecture exhibited healthy tubules that were packed closely with minimal connective tissue, and the lumen was full of mature spermatozoa. The seminal vesicle structure did not show any abnormal lesions and consisted of a single-coiled tubular branch with eosinophilic secretions in the central lumen. The prostate had a normal structure containing a folded, branching epithelial glandular architecture surrounding fibromuscular stroma, and a lumen of acinar cells containing eosinophilic secretions.

### 3.4. Fecal Gut Microbiota

**Figure 3:**
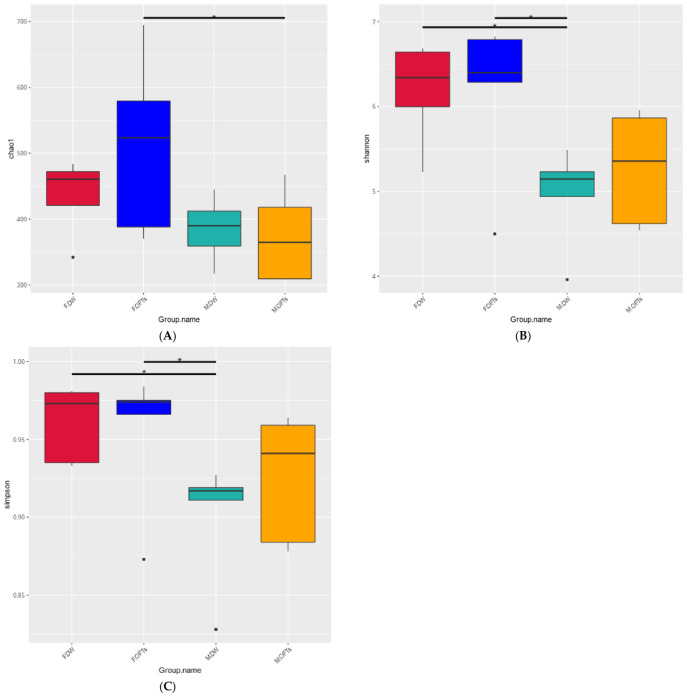
Effect of oligofructans administration on gut microbial alpha diversity in rats. Boxplots representing (**A**) Chao1 index, (**B**) Shannon index, and (**C**) Simpson index of the fecal microbiota among the four experimental groups (*n* = 5 per group): female control (F.DW; Red), female oligofructans-treated (F.OFTs; Blue), male control (M.DW; Green), and male oligofructans-treated (M.OFTs; Yellow) rats. Significant differences were assessed using the Kruskal–Wallis test. * indicates *p* < 0.05.

The alpha diversity of the gut microbiota was assessed using the Chao1, Shannon, and Simpson indices (Figure 3). In all metrics, female rats (F.DW and F.OFTs) had a much higher alpha diversity than male rats (M.DW and M.OFTs). Although oligofructans administration showed a trend toward higher richness in female rats (Chao1 index), the Shannon and Simpson indices were higher in female rats than in male rats. The effect of oligofructans was modest compared with that of the host sex.

**Figure 4:**
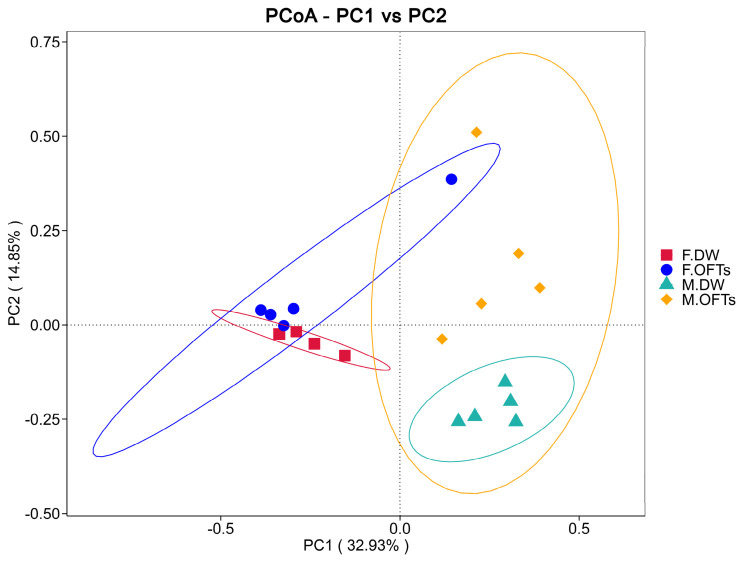
Principal Coordinates Analysis (PCoA) of gut microbial community structure based on Bray–Curtis dissimilarity. The plot visualizes the beta-diversity among four experimental groups: female control (F.DW; red squares), female oligofructans-treated (F.OFTs; blue circles), male control (M.DW; green triangles), and male oligofructans-treated (M.OFTs; yellow diamonds) rats. Each point represents an individual sample (*n* = 5 per group).

To evaluate whether the administration of oligofructans altered the overall microbial community structure, PCoA was conducted based on Bray–Curtis dissimilarity (Figure 4). The microbial communities demonstrated distinct separation, primarily influenced by host sex. In both sexes, the oligofructans-treated groups (F.OFTs and M.OFTs) exhibited distinct clustering patterns compared with those in their corresponding control groups (F.DW and M.DW). This indicated a compositional shift in the gut microbiota following oligofructans administration.

The PCoA analysis indicated that host sex was the primary factor shaping the gut microbial structure, and the microbial composition differed according to oligofructans treatment. Oligofructans administration caused a notable compositional alteration (beta diversity), such that the alpha-diversity metrics alone could not fully account for it. This structural modification indicated that oligofructans reshaped bacterial community composition through selective modulation of specific microbial taxa rather than increasing overall diversity. These findings indicate the efficacy of oligofructans as prebiotic candidates that alter gut microbial composition in a sex-dependent manner.

**Figure 5:**
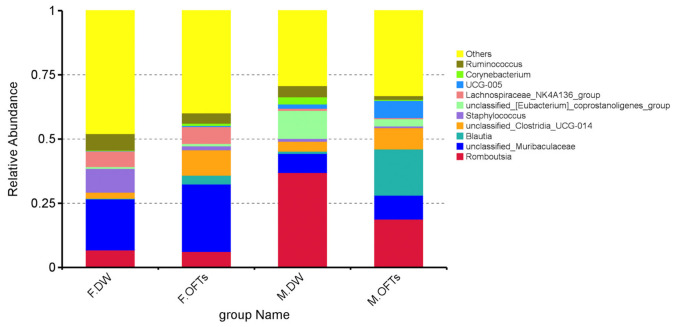
Relative abundance of bacterial genera in each experimental group. Stacked bar plots display the relative abundance of gut bacterial genera of the fecal microbiota among the four experimental groups (*n* = 5 per group): female control (F.DW), female oligofructans-treated (F.OFTs), male control (M.DW), and male oligofructans-treated (M.OFTs) rats.

Figure 5 illustrates the relative abundances of the bacterial genera in each group. In males, the control group (M.DW) was dominated by *Romboutsia*; however, its relative abundance markedly decreased in the oligofructans-treated group (M.OFTs). This reduction was accompanied by an increased abundance of unclassified *Muribaculaceae* and *Blautia*, which suggests a shift toward bacteria associated with carbohydrate fermentation. In females, the microbial composition appeared to be more stable than that in males, with only modest differences observed between the control and treated groups. The relative abundance of *Romboutsia* remained constant. Oligofructans supplementation increased the prevalence of unclassified *Muribaculaceae*, *Blautia*, and unclassified *Clostridia_UCG-014*, while decreasing *Staphylococcus* compared with that in the control group.

**Figure 6:**
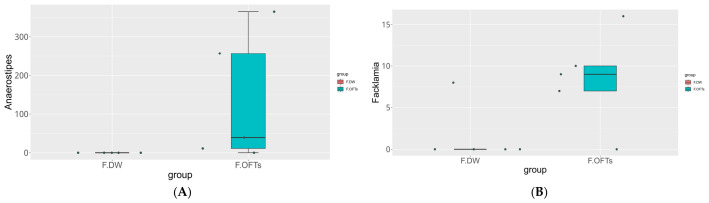

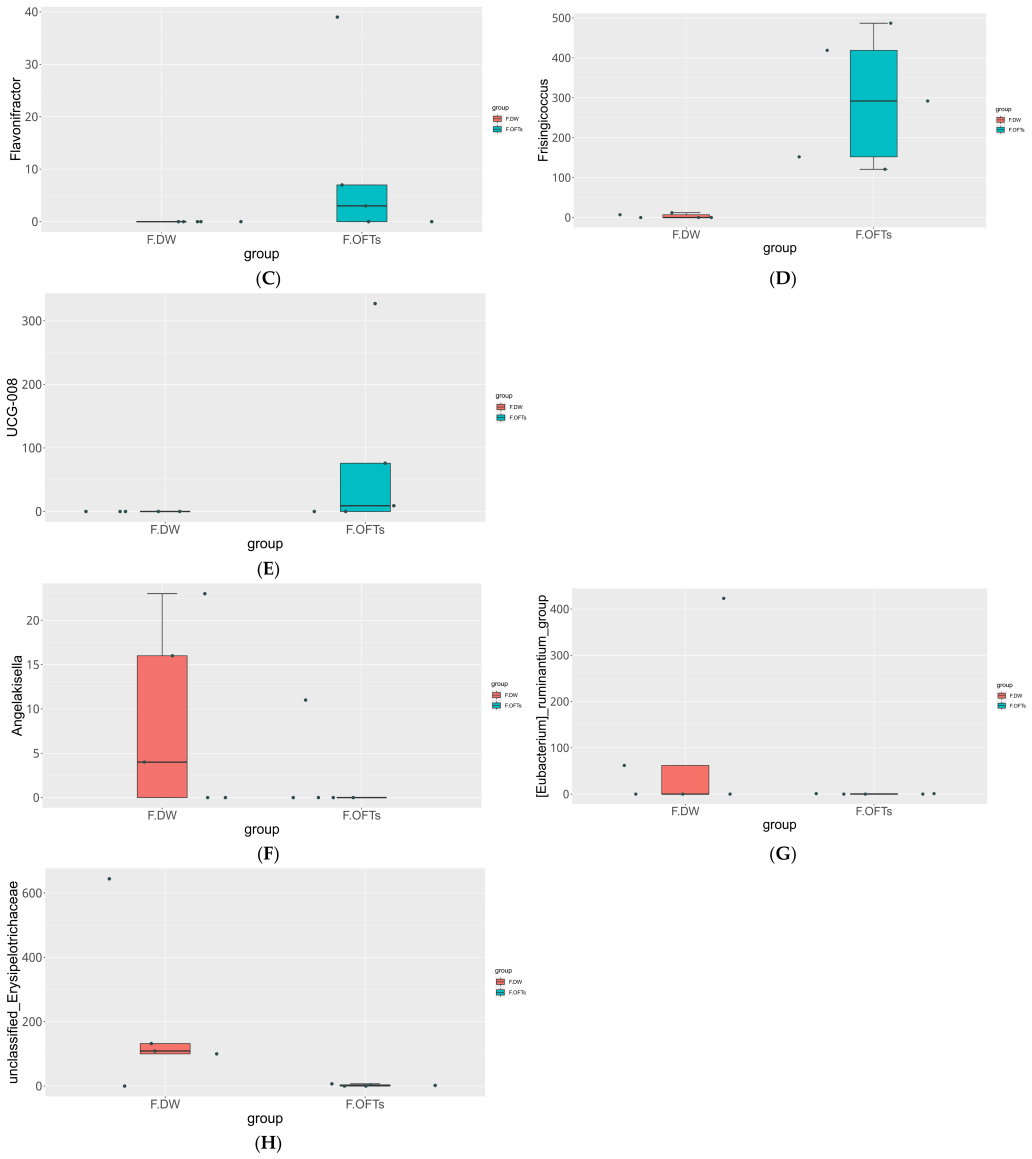
Relative abundance of differentially abundant bacteria in female rats following oligofructans treatment. Box plots showing the relative abundance of significantly altered genera in female control (F.DW) and female oligofructans-treated (F.OFTs) rats (*n* = 5 per group): (**A**) *Anaerostipes*, (**B**) *Facklamia*, (**C**) *Flavonifractor*, (**D**) *Frisingicoccus*, (**E**) *UCG-008*, (**F**) *Angelakisella*, (**G**) [*Eubacterium]_ruminantium*_group, and (**H**) unclassified_*Erysipelotrichaceae*.

Taxonomic analysis at the genus level revealed substantial alterations in some bacterial communities in female rats after oligofructans administration (Figure 6). Notably, an increase in the abundance of specific taxa in the oligofructans-treated group was observed. These taxa included *Anaerostipes*, *Facklamia*, *Flavonifractor*, *Frisingicoccus*, and *UCG-008*, which showed an increased relative abundance compared with that in the control group. In contrast, the oligofructans-treated group exhibited a pronounced decrease in the relative abundances of *[Eubacterium]_ruminantium*_group, *Angelakisella*, and unclassified_*Erysipelotrichaceae*.

**Figure 7:**
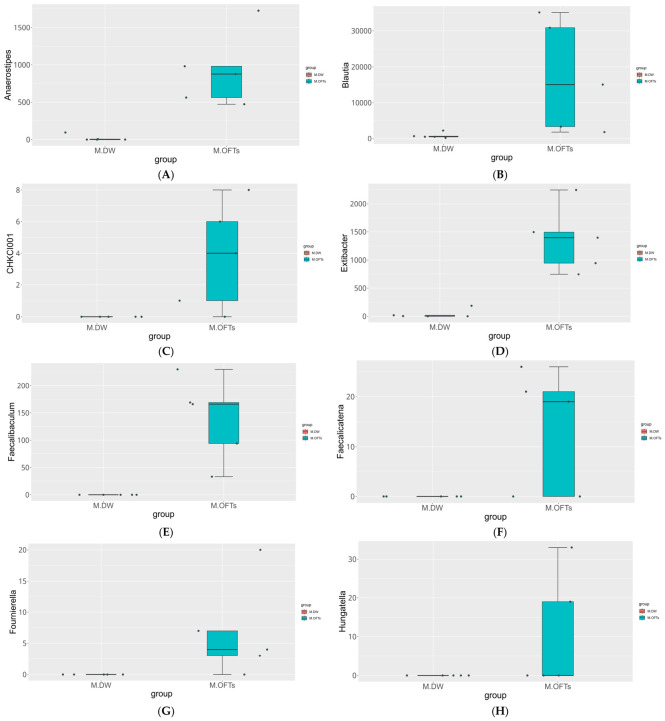

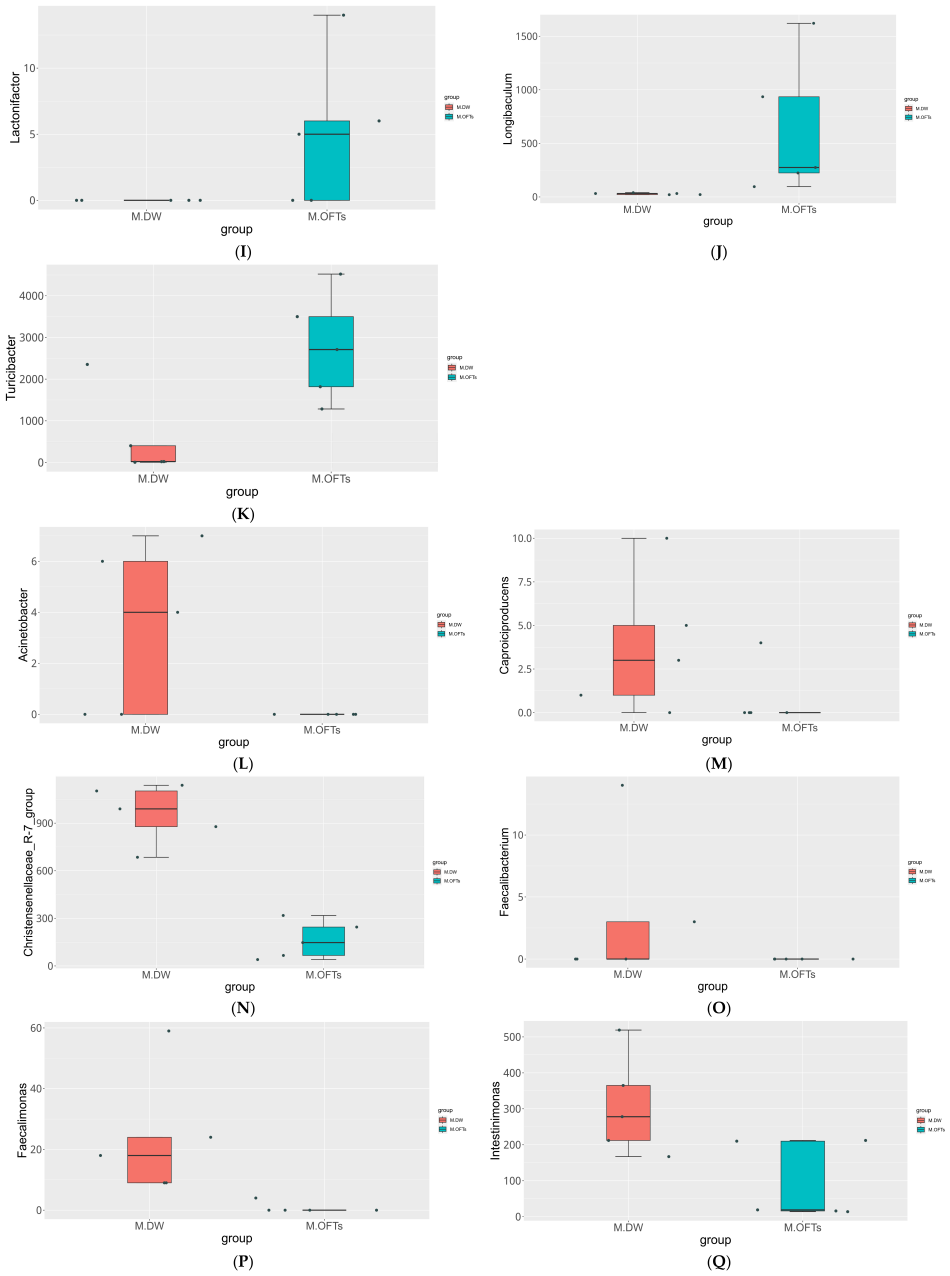

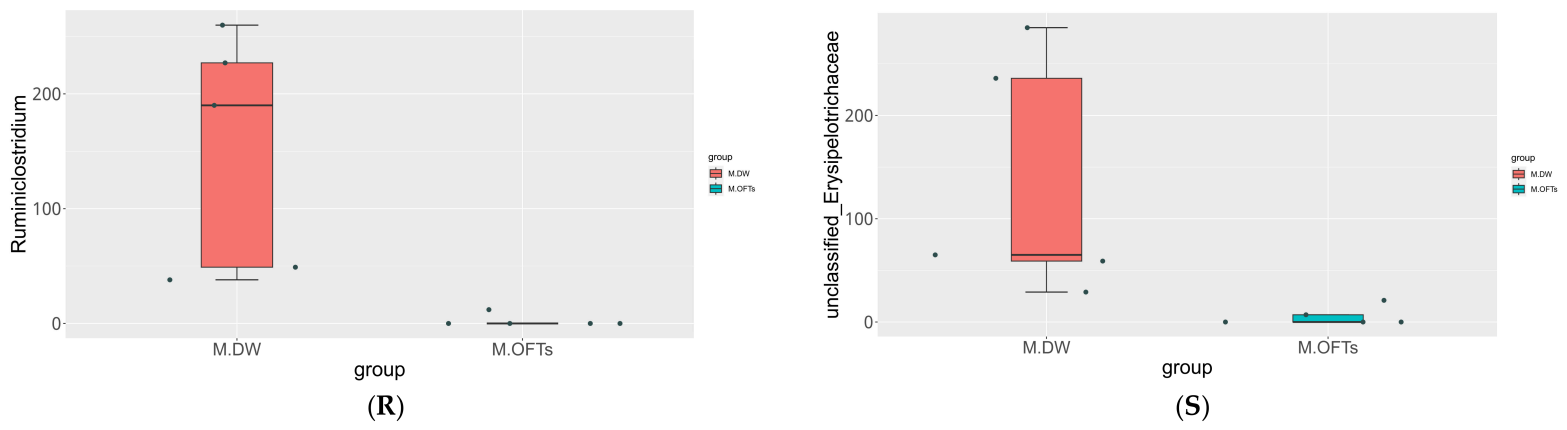
Relative abundance of differentially abundant bacteria in male rats following oligofructans treatment. Box plots showing the relative abundance of significantly altered genera in male control (M.DW) and male oligofructans-treated (M.OFTs) rats (*n* = 5 per group): (**A**) *Anaerostipes*, (**B**) *Blautia*, (**C**) *CHKCI001*, (**D**) *Extibacter*, (**E**) *Faecalibaculum*, (**F**) *Faecalicatena*, (**G**) *Fournierella*, (**H**) *Hungatella*, (**I**) *Lactonifactor*, (**J**) *Longibaculum*, (**K**) *Turicibacter*, (**L**) *Acinetobacter*, (**M**) *Caproiciproducens*, (**N**) *Christensenellaceae_R-7*_group, (**O**) *Faecalibacterium*, (**P**) *Faecalimonas*, (**Q**) *Intestinimonas*, (**R**) *Ruminiclostridium*, and (**S**) unclassified_*Erysipelotrichaceae*.

As shown in Figure 7, the analysis of bacterial genus abundance in male rats revealed that oligofructans treatment markedly altered the gut microbial community composition. Specifically, the relative abundances of several genera, including *Anaerostipes*, *Blautia*, *CHKCI001*, *Extibacter*, *Faecalibaculum*, *Faecalicatena*, *Fournierella*, *Hungatella*, *Lactonifactor*, *Longibaculum*, and *Turicibacter*, were substantially higher in the oligofructans-treated group than in the control group. Conversely, the abundances of *Acinetobacter*, *Caproiciproducens*, *Christensenellaceae_R-7*_group, *Faecalibacterium*, *Faecalimonas*, *Intestinimonas*, *Ruminiclostridium*, and unclassified_*Erysipelotrichaceae* were markedly lower in the oligofructans-treated group than in the control group. These results suggest that oligofructans administration alters the composition of specific bacterial genera in the gut microbiota of female and male rats.

## 4. Discussion

Oligofructans produced from raw sugar by *B*. *subtilis* TISTR 001 fermentation possess prebiotic properties and effectively prevent chemically induced colorectal carcinogenesis in rats [13]. Thus, to ensure the safety of these oligofructans for human use, this study aimed to assess their safety using acute and subchronic oral toxicity tests conducted over 90 days.

In the acute toxicity test, oligofructans were not toxic at a dose of 2000 mg/kg bw, and no treatment-related effects on survival, behavior, or organ morphology were observed. According to the Globally Harmonized System of Classification and Labelling of Chemicals, compounds with an LD_50_ >2000 mg/kg are classified as Category 5 or are unclassified [24]. This indicated that these oligofructans exhibited very low acute toxicity profiles.

Subchronic toxicity testing was conducted to investigate possible adverse effects of plant extracts or novel compounds following repeated exposure over a duration of time. This test was used to identify the target organ toxicity and determine the no-observed-adverse-effect level (NOAEL) [25,26,27,28]. Alterations in general behavior and body weight are regarded as crucial criteria for evaluating the early signs of toxicity caused by drugs and chemicals [29]. The current study showed that the administration of oligofructans at the highest dose of 2000 mg/kg bw tended to decrease body weight and dietary intake in male and female rats. More pronounced decreases were observed in the male satellite group. These findings may be related to the effects of prebiotic dietary fibers on satiety and energy metabolism, rather than systemic toxicity. This finding is consistent with the functional properties of inulin, which is a water-soluble dietary fiber that regulates glucose levels, reduces obesity, and enhances gastrointestinal function [30]. Furthermore, inulin enhances satiety, reduces appetite, and decreases energy intake in healthy females and decreases diabetes risk by promoting weight reduction and lowering lipid levels in people with prediabetes, independent of weight loss [31,32]. Moreover, oligofructose (Raftilose P95) supplementation contributes to weight loss and improved glucose regulation in overweight adults, independent of other lifestyle changes. Reduced ghrelin, an orexigenic peptide, and increased PYY, an anorexigenic peptide, may partially contribute to decreased energy intake [33]. Therefore, the observed reductions in body weight and food intake may reflect functional dietary fiber activity rather than adverse effects.

Evaluation of organ weight in toxicity studies is essential for assessing the safety of drugs, chemical substances, and medical devices [34]. In this study, several statistically significant changes in absolute and relative organ weights were observed; however, these changes were not consistently dose-dependent [35,36,37]. The observed alterations in the absolute weight of the spleen in females (200 mg/kg bw) and the relative weight of the pancreas in males (200 and 600 mg/kg bw) occurred without a clear dose–response relationship and were not accompanied by corresponding histopathological abnormalities. Moreover, the pronounced increases in the relative weights of the kidneys and heart in the high-dose female satellite group were not matched by the corresponding changes in their absolute weights. Interestingly, the only observed corresponding increase in absolute and relative values was in the lungs of males treated with the highest dose (2000 mg/kg bw). However, histopathological findings were used to confirm the toxic effects of oligofructans on internal organs.

The hematopoietic system is a sensitive target for toxic substances and a key indicator of physiological and pathological conditions [37]. This study found inconsistent statistically significant differences in certain parameters, including concurrent increases in RBC count and Hct, as well as a decrease in WBC count in high-dose female rats. In standard toxicological tests, biologically relevant adverse effects are generally defined by clear dose-dependent patterns [37]. However, these changes were inconsistent, sex-specific, and not accompanied by dose-dependent trends or histopathological findings. Therefore, they are considered to have limited toxicological significance under the conditions of this study.

The assessment of biochemical parameters reveals information regarding the adverse effects of a chemical substance on the function of the main organ systems, including the liver and kidneys [38]. The liver and kidneys are the primary organs responsible for the metabolism and excretion of drugs and other xenobiotics. These organs are the primary target organs that exhibit systemic adverse effects following oral drug administration [39]. Elevated serum ALT levels indicate hypertrophy and damage to hepatic tissue, whereas AST levels are a marker of liver failure and are used to evaluate muscular and cardiac disorders [37,40]. ALP primarily accumulates in the cells lining the biliary duct of the liver and is mainly used to diagnose bile duct abnormalities [37]. Bilirubin is a product of Hb degradation and plays a major role in the diagnosis, prognosis, and monitoring of liver diseases [41]. Serum total protein levels serve as a general indicator of protein status, which may indicate functional alterations in renal and hepatic function [37]. The kidneys are essential organs that are particularly susceptible to harmful substances owing to their high blood flow. Uric acid and creatinine levels are sensitive indicators of renal dysfunction [37]. Markers of liver damage, specifically AST, ALT, and ALP, along with renal function indicators, such as BUN and lipid profiles, remained entirely unaffected across all experimental groups. Furthermore, minor fluctuations in creatinine, electrolytes (including Na^+^ and K^+^), total bilirubin, and glucose were observed. However, these variations lack toxicological relevance. These results indicate that oligofructans may not cause kidney damage or have adverse effects on the erythropoietic system, liver, or renal function.

In the subchronic toxicity test, histopathological evaluation was performed to corroborate the physiological and biochemical findings. Microscopic examination of the internal organs revealed no cellular abnormalities or pathological lesions in rats administered 2000 mg/kg bw of oligofructans. Importantly, normal functional serum biochemical parameters, such as AST, ALT, and BUN, were precisely paralleled by the preserved structure of the hepatic architecture (hepatocytes and sinusoids) and renal components (glomeruli and tubules). These results showed that repeated oligofructans treatment did not cause target organ toxicity, which is supported by this structural–functional concordance. The aforementioned variations in male lung weights may be attributed to accidental changes because the pulmonary architecture did not show any related inflammatory epithelium changes or tissue remodeling. Similarly, the normal architecture of the reproductive organs, adrenal glands, and pancreas confirmed the absence of endocrine-disrupting features of oligofructans.

Analysis of the gut microbiota revealed that host sex had a stronger influence than that of dietary intervention on the gut microbiota. Sex differences in hormones, immunity, and physiology affect the colonization of gut microbes, as well as the stability of bacterial communities. The higher diversity observed in females than in males may reflect increased ecological resilience in the gut [42]. The increased alpha diversity reported in females may indicate a more robust microbial community than that in males. The current study confirmed that both species richness and evenness (Shannon and Simpson indices) were substantially higher in females than in males. Although a potential increase in microbial richness was observed within the oligofructans-treated group, the slight alteration indicated that the effects of oligofructans may be accurately assessed in the context of host sex, such as baseline microbial differences or the prevalence of particular functional taxa, rather than total species richness. The notable difference in alpha diversity across sexes emphasizes that host biological factors, especially sex, are fundamental indicators of the gut microbial community.

The study findings indicate that oligofructans supplementation successfully altered the gut microbiota composition in a sex-specific manner. In males, oligofructans treatment induced a distinct shift from *Romboutsia* prevalence to carbohydrate-fermenting taxa, including unclassified *Muribaculaceae* and *Blautia*. In contrast, the female microbiota demonstrated enhanced resilience, characterized by stable microbial profiles, an increase in beneficial fermenters, and a decrease in potentially opportunistic *Staphylococcus*. Members of *Muribaculaceae* are recognized as carbohydrate-fermenting bacteria that produce short-chain fatty acids [43]. Although direct evidence linking *Muribaculaceae* to oligofructans or FOSs remains limited, their increased abundance suggests their potential role in dietary fiber utilization. Further studies are required to clarify these interactions and their underlying mechanisms. Consistent with a previous study [13], oligofructans administration increased the abundance of *Blautia* spp., which are key FOS-utilizing bacteria, thereby validating the findings of the current study. Additionally, oligofructans administration altered the abundance of unclassified *Clostridia*_UCG-014. Certain *Clostridia* species ferment oligosaccharides, including FOSs, galactooligosaccharides, and raffinose, leading to the production of short-chain fatty acids (SCFAs) [44]. However, further taxonomic characterization is required to identify the specific *Clostridia* species affected in this study and to clarify their functional roles.

Analysis of bacterial genus abundance in female and male rats demonstrated that oligofructans administration substantially modulated the gut microbiota, thereby exhibiting a dual effect: enhancement of beneficial taxa and suppression of taxa associated with inflammation or metabolic dysfunction. In this study, an increase in the prevalence of *Anaerostipes* was observed in oligofructans-treated male and female rats. *Anaerostipes* is a member of the *Lachnospiraceae* family in the phylum Bacillota (previously known as Firmicutes). *Anaerostipes*, along with other *Lachnospiraceae* taxa, such as *Coprococcus* and *Roseburia*, ferments dietary fibers and resistant starches into SCFAs [45]. A low abundance of *Anaerostipes* is frequently found in gastrointestinal disorders, such as colorectal cancer, inflammatory bowel disease, and irritable bowel syndrome [46,47,48]. The enhanced *Anaerostipes* abundance in the oligofructans-treated group confirms a possible prebiotic effect.

Moreover, the study findings indicated a markedly increased abundance of *Flavonifractor* in oligofructans-treated female rats. *Flavonifractor*, particularly *Flavonifractor plautii*, is the most widely studied species and a well-known member of the human gut microbiota that contribute greatly to the biotransformation of dietary flavonoids [49,50]. This enrichment is particularly noteworthy when considering the oligofructans production process used in this study. Oligofructans generated via the fermentation of raw sugars may retain the profile of plant-derived phytochemicals, including flavonoids. In particular, *F. plautii* specializes in the metabolism of these specific polyphenolic compounds; therefore, the microbe likely grows because these substrates are available in raw sugar-based prebiotics. This suggests that the remaining phytochemicals selectively support the growth of flavonoid-degrading gut bacteria.

In addition, the relative abundance of various taxa, including *Blautia*, *Faecalibaculum*, *Hungatella*, and *Turicibacter*, was markedly elevated in male rats treated with oligofructans. *Blautia* is a member of the *Lachnospiraceae* family that exhibits probiotic properties and is commonly found in the feces and intestines of mammals. *Blautia* has attracted considerable interest since its discovery owing to its role in reducing inflammatory and metabolic disorders, as well as its antimicrobial properties against particular pathogens [51,52]. The composition and alterations of the *Blautia* population inside the gut are associated with factors, such as host age, geography, diet, genotype, health, disease status, and other physiological conditions [52,53,54,55]. Moreover, a pronounced increase in the abundance of *Blautia* was observed in the feces of mice administered with FOS [54]. The current study demonstrated that oligofructans, which contain FOS, could be used as prebiotics to enhance the abundance of *Blautia*, which would improve its potential to support gut health in the host. Moreover, the study findings indicate a pronounced increase in the genus *Faecalibaculum* following oligofructans treatment. *Faecalibaculum*, especially *Faecalibaculum rodentium*, is increasingly recognized for its probiotic potential [56,57]. *Faecalibaculum* is commonly recognized as an indicator of a healthy gut microbiome, contributes to maintaining gastrointestinal homeostasis, and is often decreased in colorectal cancer [57]. In this study, oligofructans promoted the growth of *Faecalibaculum* bacteria, which may aid in maintaining gastrointestinal homeostasis. The study findings indicated that oligofructans administration may provide benefits by restoring these essential populations.

Moreover, a substantial decrease in the abundance of unclassified *Erysipelotrichaceae* was observed in both male and female rats of the oligofructans-treated group. *Erysipelotrichaceae* abundance often increases in response to high-fat or Western diets in animal models and in individuals with obesity [58,59]. Beyond metabolic problems, the clinical significance of *Erysipelotrichaceae* extends to inflammation-related gastrointestinal illnesses, as indicated by their increased abundance in patients with colorectal cancer and animal models of chemically induced colon cancer [60,61].

The current study revealed a decrease in the relative abundance of *Christensenellaceae_R-7*_group, *Faecalibacterium*, *Intestinimonas*, and *Faecalimonas* in male rats treated with oligofructans. This reduction in *Faecalimonas* suggests a beneficial effect. *Faecalimonas*, such as *Faecalimonas umbilicate*, are linked to inflammatory processes and disease onset. This genus has also been implicated in Crohn’s disease models [62], although *Faecalibacterium* and *Intestinimonas* are widely recognized for their important contributions to butyrate production and gut health [63,64,65], and the *Christensenellaceae_R-7*_group is prevalent in individuals with healthy metabolic profiles and is negatively correlated with obesity and metabolic syndrome [66,67]. Their decline in the current study may be associated with reconfiguration of the microbial community.

The study results suggest that oligofructans administration induces an alteration in the gut microbial community rather than a uniform increase in overall diversity. This shift is characterized by an altered abundance of functional microbial taxa, including the enrichment of potentially beneficial commensal bacteria and the reduction of taxa associated with inflammation or metabolic dysfunction. Collectively, these findings support the potential prebiotic effects of oligofructans and highlight host sex as an important determinant of gut microbiota composition and responsiveness to dietary interventions.

From a translational perspective, this study used oral administration and repeated dosing in rats, which is relevant to the expected human exposure scenario for oligofructans as a dietary ingredient consumed regularly. Nevertheless, extrapolation from rats to humans should be made cautiously. Human-equivalent intake levels should be derived using appropriate interspecies dose translation approaches, including body-surface-area-based scaling, together with uncertainty factors, and the potential for interindividual variability in humans should be considered. In addition, while microbiota modulation in rats is observed with oligofructans administration, the magnitude and direction of microbiome changes may differ in humans due to differences in baseline diet, host physiology, and gut microbiome composition. Therefore, confirmatory clinical studies are warranted to evaluate tolerability and gastrointestinal outcomes and to verify microbiome-related effects in humans, ideally including relevant functional readouts, including SCFA measurements, to support mechanistic interpretation.

Limitations of this study should be acknowledged. First, although the rat model with oral repeated administration provides useful information for safety and microbiota-related responses, extrapolation to humans remains uncertain due to species-specific differences in physiology and gut microbiome composition. Second, short-chain fatty acids (SCFAs) were not directly measured; therefore, any interpretation regarding SCFA production or SCFA-mediated mechanisms remains inferential. Future studies incorporating direct SCFA quantification and confirmatory assessments in humans will be important to strengthen translational interpretation.

## 5. Conclusions

In the acute toxicity test, an oral dose of 2000 mg/kg of oligofructans produced from raw sugar fermentation by *B*. *subtilis* TISTR001 resulted in no treatment-related signs of toxicity or mortality, or any gross abnormalities on examination of all internal organs. Thus, the LD_50_ of the oligofructans was >2000 mg/kg bw. In the oral subchronic toxicity study, daily oligofructans dosages of 200, 600, and 2000 mg/kg for 90 days did not cause lethality or toxic clinical symptoms in rats of either sex. Furthermore, no treatment-related adverse effects of oligofructans on the hematological and biochemical parameters or organ histopathology were observed in the treatment and satellite groups. Hence, the NOAEL of oligofructans under the study’s test conditions was confirmed as 2000 mg/kg/day. Moreover, oligofructans modulated the gut microbiota by promoting the growth of potentially beneficial commensal bacteria and reducing taxa associated with inflammation or metabolic dysfunction. However, further studies are required to confirm these microbiome-related changes in humans.

## Figures and Tables

**Table 1:** Compositions of oligofructans derived from raw sugar fermentation by *B. subtilis* TISTR 001.

Compositions	Content (% *w*/*w*)
Sugars composition	
Sucrose	ND
Fructose	1.43 ± 0.02
Glucose	2.75 ± 0.01
Short-chain FOSs	
1-Kestose	0.07 ± 0.03
Nystose	0.11 ± 0.01
1-Fructofuranosyl-D-nystose	ND
Total polymer as free fructose	71.24 ± 0.13

Values are expressed as mean ± S.D. ND: not detected.

**Table 2:** The body weight and diet and water consumptions of the female rats.

Groups	Oligofructans (mg/kg bw)	Body Weight (g)		Consumption	
		Initial	Final	Diet (g/Rat/Day)	Water (mL/Rat/Day)
Treatment	0	182 ± 3	326 ± 7	16.4 ± 0.2	24.8 ± 0.6
	200	179 ± 2	314 ± 4	16.0 ± 0.3	23.7 ± 0.5
	600	180 ± 3	317 ± 6	16.1 ± 0.2	26.2 ± 0.6
	2000	178 ± 3	317 ± 10	15.8 ± 0.2	25.5 ± 0.6
Satellite	0	186 ± 6	354 ± 17	17.0 ± 0.3	27.9 ± 0.7
	2000	187 ± 3	338 ± 10	15.9 ± 0.3 **	28.3 ± 0.6

Values are expressed as mean ± SEM. *n* = 10 per group (treatment groups), *n* = 5 per group (satellite groups. ** Significant difference compared with the satellite control group at *p* < 0.05.

**Table 3:** The body weight and diet and water consumptions of the male rats.

Groups	Oligofructans (mg/kg bw)	Body Weight (g)		Consumption	
		Initial	Final	Diet (g/Rat/Day)	Water (mL/Rat/Day)
Treatment	0	255 ± 4	601 ± 17	25.6 ± 0.2	44.0 ± 1.6
	200	255 ± 4	607 ± 13	25.8 ± 0.2	46.5 ± 2.2
	600	256 ± 2	607 ± 13	24.9 ± 0.2	48.1 ± 2.1
	2000	254 ± 3	585 ± 9	24.3 ± 0.2 *	41.7 ± 1.3
Satellite	0	260 ± 9	716 ± 5	26.1 ± 0.3	54.1 ± 2.4
	2000	250 ± 3	671 ± 13 **	24.6 ± 0.2 **	41.0 ± 1.4 **

Values are expressed as mean ± SEM. *n* = 10 per group (treatment groups), *n* = 5 per group (satellite groups). * Significant difference compared with the control group at *p* < 0.05. ** Significant difference compared with the satellite control group at *p* < 0.05.

**Table 4:** The absolute and relative organ weights of the female rats.

Organs	Treatment				Satellite	
	Oligofructans (mg/kg bw)				Oligofructans (mg/kg bw)	
	0	200	600	2000	0	2000
Absolute (g)						
Liver	9.44 ± 0.40	9.59 ± 0.25	10.41 ± 0.45	9.76 ± 0.38	10.12 ± 0.42	9.68 ± 0.43
Spleen	0.49 ± 0.02	0.42 ± 0.02 *	0.46 ± 0.01	0.50 ± 0.02	0.47 ± 0.05	0.47 ± 0.03
Kidney	1.95 ± 0.07	1.96 ± 0.05	2.03 ± 0.06	1.97 ± 0.06	2.01 ± 0.07	2.05 ± 0.07
Lung	1.40 ± 0.09	1.34 ± 0.03	1.39 ± 0.04	1.42 ± 0.02	1.42 ± 0.05	1.39 ± 0.07
Heart	0.87 ± 0.03	0.85 ± 0.02	0.87 ± 0.02	0.86 ± 0.03	0.94 ± 0.03	0.97 ± 0.02
Thymus	0.33 ± 0.03	0.30 ± 0.02	0.32 ± 0.01	0.33 ± 0.03	0.28 ± 0.04	0.26 ± 0.04
Pancreas	0.74 ± 0.07	0.76 ± 0.04	0.73 ± 0.04	0.70 ± 0.03	0.65 ± 0.04	0.64 ± 0.02
Brain	1.77 ± 0.04	1.78 ± 0.03	1.82 ± 0.02	1.71 ± 0.03	1.75 ± 0.07	1.79 ± 0.02
Stomach	1.32 ± 0.04	1.38 ± 0.06	1.31 ± 0.07	1.36 ± 0.05	1.45 ± 0.05	1.44 ± 0.08
Ovary	0.156 ± 0.008	0.132 ± 0.007	0.137 ± 0.007	0.153 ± 0.012	0.136 ± 0.016	0.133 ± 0.011
Adrenal gland	0.058 ± 0.003	0.065 ± 0.003	0.063 ± 0.005	0.059 ± 0.004	0.068 ± 0.004	0.075 ± 0.005
Relative (g/100 g bw)						
Liver	2.89 ± 0.08	3.06 ± 0.11	3.28 ± 0.13	3.08 ± 0.06	2.86 ± 0.06	2.86 ± 0.07
Spleen	0.15 ± 0.01	0.13 ± 0.01	0.15 ± 0.01	0.16 ± 0.00	0.13 ± 0.01	0.14 ± 0.01
Kidney	0.60 ± 0.01	0.63 ± 0.02	0.64 ± 0.01	0.62 ± 0.01	0.57 ± 0.01	0.61 ± 0.01 **
Lung	0.43 ± 0.02	0.43 ± 0.01	0.44 ± 0.01	0.45 ± 0.01	0.41 ± 0.02	0.41 ± 0.02
Heart	0.27 ± 0.01	0.27 ± 0.01	0.28 ± 0.01	0.27 ± 0.01	0.27 ± 0.01	0.29 ± 0.00 **
Thymus	0.10 ± 0.01	0.09 ± 0.01	0.10 ± 0.00	0.10 ± 0.01	0.08 ± 0.01	0.08 ± 0.01
Pancreas	0.23 ± 0.02	0.24 ± 0.01	0.23 ± 0.01	0.22 ± 0.01	0.19 ± 0.01	0.19 ± 0.01
Brain	0.55 ± 0.01	0.57 ± 0.01	0.58 ± 0.01	0.54 ± 0.01	0.50 ± 0.02	0.53 ± 0.01
Stomach	0.41 ± 0.01	0.44 ± 0.02	0.41 ± 0.02	0.43 ± 0.01	0.41 ± 0.02	0.43 ± 0.02
Ovary	0.048 ± 0.003	0.042 ± 0.002	0.044 ± 0.003	0.049 ± 0.004	0.039 ± 0.006	0.040 ± 0.004
Adrenal gland	0.018 ± 0.001	0.021 ± 0.001	0.020 ± 0.002	0.019 ± 0.001	0.020 ± 0.002	0.022 ± 0.002

Values are expressed as mean ± SEM. *n* = 10 per group (treatment groups), *n* = 5 per group (satellite groups). * Significant difference compared with the control group at *p* < 0.05. ** Significant difference compared with the satellite control group at *p* < 0.05.

**Table 5:** The absolute and relative organ weights of the male rats.

Organs	Treatment				Satellite	
	Oligofructans (mg/kg bw)				Oligofructans (mg/kg bw)	
	0	200	600	2000	0	2000
Absolute (g)						
Liver	18.10 ± 0.83	19.77 ± 0.85	18.07 ± 0.54	17.82 ± 0.37	22.70 ± 0.72	21.18 ± 0.86
Spleen	0.76 ± 0.02	0.69 ± 0.02	0.72 ± 0.03	0.75 ± 0.03	0.91 ± 0.06	0.81 ± 0.04
Kidney	3.72 ± 0.12	3.81 ± 0.12	3.64 ± 0.06	3.72 ± 0.10	4.45 ± 0.06	4.30 ± 0.12
Lung	1.61 ± 0.13	1.97 ± 0.10	1.87 ± 0.07	2.37 ± 0.20 *	2.08 ± 0.06	2.12 ± 0.08
Heart	1.56 ± 0.05	1.55 ± 0.06	1.51 ± 0.06	1.55 ± 0.05	1.78 ± 0.04	1.65 ± 0.06
Thymus	0.37 ± 0.04	0.32 ± 0.03	0.36 ± 0.01	0.37 ± 0.03	0.24 ± 0.02	0.34 ± 0.04
Pancreas	1.08 ± 0.06	0.93 ± 0.05	0.89 ± 0.06	1.07 ± 0.08	1.16 ± 0.09	1.02 ± 0.08
Brain	1.92 ± 0.03	1.83 ± 0.06	1.90 ± 0.06	1.84 ± 0.05	2.09 ± 0.06	2.05 ± 0.05
Testis	3.87 ± 0.09	3.73 ± 0.06	3.92 ± 0.06	3.88 ± 0.09	3.94 ± 0.09	3.84 ± 0.09
Prostate	0.58 ± 0.05	0.69 ± 0.07	0.66 ± 0.06	0.71 ± 0.07	0.78 ± 0.09	0.72 ± 0.07
Stomach	1.87 ± 0.04	1.78 ± 0.10	1.87 ± 0.07	1.89 ± 0.07	2.15 ± 0.06	2.09 ± 0.05
Epididymis	1.45 ± 0.09	1.35 ± 0.05	1.38 ± 0.04	1.35 ± 0.07	1.63 ± 0.06	1.46 ± 0.06
Seminal vesicle	1.90 ± 0.12	1.74 ± 0.08	1.84 ± 0.10	2.15 ± 0.08	2.40 ± 0.17	2.27 ± 0.11
Adrenal gland	0.061 ± 0.003	0.067 ± 0.004	0.064 ± 0.004	0.067 ± 0.004	0.068 ± 0.002	0.065 ± 0.003
Relative (g/100 g bw)						
Liver	3.00 ± 0.09	3.25 ± 0.08	2.98 ± 0.06	3.05 ± 0.03	3.17 ± 0.10	3.15 ± 0.09
Spleen	0.13 ± 0.00	0.11 ± 0.00	0.12 ± 0.00	0.13 ± 0.00	0.13 ± 0.01	0.12 ± 0.01
Kidney	0.62 ± 0.01	0.63 ± 0.02	0.60 ± 0.01	0.64 ± 0.01	0.62 ± 0.01	0.64 ± 0.02
Lung	0.27 ± 0.03	0.33 ± 0.02	0.31 ± 0.01	0.40 ± 0.03 *	0.29 ± 0.01	0.32 ± 0.01
Heart	0.26 ± 0.01	0.26 ± 0.01	0.25 ± 0.01	0.26 ± 0.01	0.25 ± 0.00	0.25 ± 0.01
Thymus	0.06 ± 0.01	0.05 ± 0.01	0.06 ± 0.00	0.06 ± 0.01	0.03 ± 0.00	0.05 ± 0.01 **
Pancreas	0.18 ± 0.01	0.15 ± 0.01 *	0.15 ± 0.01 *	0.18 ± 0.01	0.16 ± 0.01	0.15 ± 0.01
Brain	0.32 ± 0.01	0.30 ± 0.01	0.31 ± 0.01	0.31 ± 0.01	0.29 ± 0.01	0.31 ± 0.00
Testis	0.65 ± 0.02	0.62 ± 0.02	0.65 ± 0.02	0.66 ± 0.01	0.55 ± 0.01	0.57 ± 0.01
Prostate	0.10 ± 0.01	0.11 ± 0.01	0.11 ± 0.01	0.12 ± 0.01	0.11 ± 0.01	0.11 ± 0.01
Stomach	0.31 ± 0.01	0.29 ± 0.01	0.31 ± 0.01	0.32 ± 0.01	0.30 ± 0.01	0.31 ± 0.01
Epididymis	0.24 ± 0.01	0.22 ± 0.01	0.23 ± 0.01	0.23 ± 0.01	0.23 ± 0.01	0.22 ± 0.01
Seminal vesicle	0.32 ± 0.02	0.29 ± 0.01	0.30 ± 0.02	0.37 ± 0.01	0.34 ± 0.02	0.34 ± 0.02
Adrenal gland	0.010 ± 0.001	0.011 ± 0.001	0.011 ± 0.001	0.011 ± 0.001	0.010 ± 0.000	0.010 ± 0.000

Values are expressed as mean ± SEM. *n* = 10 per group (treatment groups), *n* = 5 per group (satellite groups). * Significant difference compared with the control group at *p* < 0.05. ** Significant difference compared with the satellite control group at *p* < 0.05.

**Table 6:** Hematological parameters of the female rats.

Hematological Parameters	Treatment				Satellite		Reference Value ^a^
	Oligofructans (mg/kg bw)				Oligofructans (mg/kg bw)		
	0	200	600	2000	0	2000	
RBC (×10^6^/µL)	8.26 ± 0.08	8.31 ± 0.06	8.13 ± 0.08	8.62 ± 0.13 *	7.99 ± 0.10	7.98 ± 0.23	7.23–8.11
Hb (g/dL)	15.46 ± 0.22	15.41 ± 0.13	15.22 ± 0.12	16.11 ± 0.30	14.86 ± 0.30	15.08 ± 0.38	13.50–15.50
Hct (%)	46.27 ± 0.61	46.53 ± 0.34	45.84 ± 0.49	48.50 ± 0.76 *	43.08 ± 1.11	44.12 ± 1.36	39.60–45.90
MCV (fL)	55.98 ± 0.41	56.04 ± 0.51	56.35 ± 0.40	56.29 ± 0.50	53.88 ± 0.71	55.20 ± 0.39	53.50–58.30
MCH (pg)	18.71 ± 0.14	18.55 ± 0.18	18.74 ± 0.17	18.69 ± 0.23	18.62 ± 0.16	18.90 ± 0.11	18.50–19.50
MCHC (g/dL)	33.43 ± 0.18	33.11 ± 0.12	33.21 ± 0.23	33.21 ± 0.14	34.50 ± 0.29	34.22 ± 0.21	-
RDW-CV (%)	12.77 ± 0.14	12.66 ± 0.12	12.72 ± 0.14	12.58 ± 0.13	12.08 ± 0.21	12.06 ± 0.10	-
PLT (×10^3^ cell/µL)	1081 ± 36	1112 ± 32	1021 ± 33	1107 ± 30	1040 ± 33	929 ± 26 **	787–1021
WBC (×10^3^ cell/µL)	7.66 ± 0.64	6.11 ± 0.42	6.61 ± 0.32	5.56 ± 0.50 *	7.03 ± 0.40	5.58 ± 0.53	7.00–10.69
Neu (%)	12.74 ± 1.08	16.52 ± 1.08	15.94 ± 0.93	14.07 ± 1.62	12.04 ± 0.90	14.50 ± 1.35	7.70–14.20
Lym (%)	77.45 ± 1.72	71.39 ± 1.72	72.34 ± 1.95	76.03 ± 2.55	76.42 ± 1.32	73.26 ± 2.78	80.60–87.00
Mono (%)	8.04 ± 0.66	9.87 ± 0.90	9.98 ± 1.19	8.63 ± 1.12	9.98 ± 0.81	10.38 ± 1.67	1.40–3.30
Eos (%)	1.71 ± 0.15	2.20 ± 0.21	1.72 ± 0.09	1.27 ± 0.11	1.54 ± 0.16	1.86 ± 0.29	0.60–1.60
Baso (%)	0.06 ± 0.03	0.02 ± 0.01	0.02 ± 0.01	0.00 ± 0.00 *	0.02 ± 0.02	0.00 ± 0.00	0.20–0.50

Values are expressed as mean ± SEM. *n* = 10 per group (treatment groups), *n* = 5 per group (satellite groups). * Significant difference compared with the control group at *p* < 0.05. ** Significant difference compared with the satellite control group at *p* < 0.05. ^a^ Reference value of 13–22 weeks of Age [23].

**Table 7:** Hematological parameters of the male rats.

Hematological Parameters	Treatment				Satellite		Reference Value ^a^
	Oligofructans (mg/kg bw)				Oligofructans (mg/kg bw)		
	0	200	600	2000	0	2000	
RBC (×10^6^/µL)	9.71 ± 0.08	9.51 ± 0.07	9.67 ± 0.07	9.68 ± 0.08	9.00 ± 0.15	9.04 ± 0.10	7.89–8.90
Hb (g/dL)	17.10 ± 0.14	16.67 ± 0.07	17.04 ± 0.16	16.95 ± 0.13	15.96 ± 0.21	16.16 ± 0.18	14.70–16.60
Hct (%)	52.20 ± 0.60	50.30 ± 0.33	51.21 ± 0.55	50.91 ± 0.32	47.14 ± 0.79	47.50 ± 0.64	28.30–49.20
MCV (fL)	53.75 ± 0.28	52.92 ± 0.48	52.92 ± 0.36	52.59 ± 0.31	52.44 ± 0.82	52.58 ± 0.53	51.70–58.40
MCH (pg)	17.62 ± 0.14	17.55 ± 0.17	17.62 ± 0.13	17.53 ± 0.13	17.76 ± 0.28	17.90 ± 0.19	17.70–19.00
MCHC (g/dL)	32.76 ± 0.21	33.14 ± 0.18	33.29 ± 0.15	33.30 ± 0.13	33.88 ± 0.18	34.04 ± 0.16	-
RDW-CV (%)	13.59 ± 0.14	13.65 ± 0.09	13.30 ± 0.09	13.18 ± 0.10	14.08 ± 0.21	13.76 ± 0.33	-
PLT (×10^3^ cell/µL)	1140 ± 34	1116 ± 48	1066 ± 24	1018 ± 32	1013 ± 31	972 ± 66	765–1029
WBC (×10^3^ cell/µL)	12.54 ± 0.54	13.88 ± 0.71	13.19 ± 0.89	12.87 ± 0.55	11.83 ± 0.80	10.77 ± 0.36	9.78–12.90
Neu (%)	15.67 ± 1.28	14.64 ± 1.30	15.36 ± 0.84	17.42 ± 2.41	13.80 ± 0.99	17.34 ± 1.10 **	9.00–13.60
Lym (%)	75.72 ± 1.65	73.87 ± 1.68	75.40 ± 1.30	71.38 ± 2.64	73.58 ± 1.37	69.44 ± 1.69	80.10–87.10
Mono (%)	6.92 ± 0.59	9.63 ± 0.92	7.27 ± 0.61	9.14 ± 1.22	10.84 ± 0.70	11.44 ± 0.59	1.10–4.10
Eos (%)	1.38 ± 0.10	1.50 ± 0.09	1.62 ± 0.16	1.67 ± 0.15	1.44 ± 0.13	1.44 ± 0.12	0.70–2.00
Baso (%)	0.31 ± 0.04	0.36 ± 0.07	0.35 ± 0.08	0.39 ± 0.05	0.34 ± 0.05	0.34 ± 0.07	0.30–0.50

Values are expressed as mean ± SEM. *n* = 10 per group (treatment groups), *n* = 5 per group (satellite groups). ** Significant difference compared with the satellite control group at *p* < 0.05. ^a^ Reference value of 13–22 weeks of Age [23].

**Table 8:** Biochemical and electrolyte parameters of the female rats.

Biochemical Parameters	Treatment				Satellite		Reference Value ^a^
	Oligofructans (mg/kg bw)				Oligofructans (mg/kg bw)		
	0	200	600	2000	0	2000	
Glucose (mg/dL)	216.8 ± 14.6	202.3 ± 17.7	212.9 ± 10.0	223.6 ± 17.8	173.4 ± 12.8	247.8 ± 21.5 **	120.0–186.0
BUN (mg/dL)	22.9 ± 0.8	22.1 ± 0.7	22.0 ± 0.8	22.6 ± 1.6	13.8 ± 0.7	17.6 ± 1.9	11.0–17.0
Creatinine (mg/dL)	0.9 ± 0.0	1.0 ± 0.0 *	1.0 ± 0.0	1.0 ± 0.0	0.9 ± 0.0	0.9 ± 0.0	0.4–0.7
AST (U/L)	108.3 ± 7.6	148.8 ± 12.0	172.4 ± 37.9	116.1 ± 9.2	138.2 ± 56.6	77.2 ± 9.3	72.0–116.0
ALT (U/L)	58.0 ± 10.2	69.4 ± 6.6	82.5 ± 23.5	58.7 ± 2.4	72.8 ± 33.9	54.4 ± 10.0	25.0–45.0
ALP (U/L)	33.0 ± 1.9	31.1 ± 1.7	29.2 ± 1.0	31.0 ± 1.4	25.0 ± 3.8	26.6 ± 2.7	65.0–117.0
Total protein (g/dL)	8.8 ± 0.2	9.3 ± 0.2	9.1 ± 0.2	9.2 ± 0.2	8.0 ± 0.2	8.0 ± 0.2	6.4–7.5
Albumin (g/dL)	4.6 ± 0.1	4.8 ± 0.1	4.7 ± 0.1	4.8 ± 0.0	4.6 ± 0.1	4.6 ± 0.1	3.5–5.3
Globulin (g/dL)	4.2 ± 0.1	4.4 ± 0.1	4.4 ± 0.1	4.4 ± 0.1	3.4 ± 0.2	3.4 ± 0.2	-
Total bilirubin (mg/dL)	0.1 ± 0.0	0.1 ± 0.0	0.1 ± 0.0	0.1 ± 0.0	0.2 ± 0.0	0.2 ± 0.0	0.2–2.0
Direct bilirubin (mg/dL)	0.1 ± 0.0	0.1 ± 0.0	0.1 ± 0.0	0.1 ± 0.0	0.1 ± 0.0	0.1 ± 0.0	-
Cholesterol (mg/dL)	90.5 ± 4.1	102.0 ± 6.0	90.7 ± 4.4	85.0 ± 3.9	103.8 ± 4.9	115.8 ± 7.8	66.0–97.0
Triglyceride (mg/dL)	238.1 ± 34.7	217.8 ± 23.1	199.2 ± 19.2	198.9 ± 26.6	250.2 ± 58.3	236.6 ± 29.3	51.0–75.0
HDL-C (mg/dL)	63.4 ± 4.2	76.9 ± 5.1	68.0 ± 3.5	62.8 ± 1.9	57.5 ± 4.2	65.9 ± 5.9	-
LDL-C (mg/dL)	10.2 ± 0.6	12.6 ± 1.4	9.7 ± 0.9	9.2 ± 0.8	8.0 ± 0.8	9.4 ± 1.3	-
Uric acid (mg/dL)	5.6 ± 0.7	6.7 ± 0.5	8.3 ± 1.0	8.7 ± 0.5 *	5.4 ± 0.4	6.6 ± 0.7	-
Na^+^ (mmol/L)	145.6 ± 0.4	146.3 ± 0.6	143.1 ± 0.8 *	144.3 ± 0.7	145.2 ± 0.5	145.3 ± 0.6	141.0–148.0
K^+^ (mmol/L)	5.7 ± 0.2	7.4 ± 0.5	10.1 ± 1.6 *	8.8 ± 0.8	5.7 ± 0.3	6.1 ± 0.4	4.3–5.9
Cl^−^ (mmol/L)	101.4 ± 0.4	101.3 ± 0.4	101.1 ± 0.4	100.7 ± 0.5	98.0 ± 0.3	97.7 ± 0.3	101.0–108.0
TCO_2_ (mmol/L)	26.0 ± 1.0	26.3 ± 0.4	24.5 ± 0.6	24.4 ± 0.5	27.5 ± 1.0	26.6 ± 1.3	-

Values are expressed as mean ± SEM. *n* = 10 per group (treatment groups), *n* = 5 per group (satellite groups). * Significant difference compared with the control group at *p* < 0.05. ** Significant difference compared with the satellite control group at *p* < 0.05. ^a^ Reference value of 13–22 weeks of Age [23].

**Table 9:** Biochemical and electrolyte parameters of the male rats.

Biochemical Parameters	Treatment				Satellite		Reference Value ^a^
	Oligofructans (mg/kg bw)				Oligofructans (mg/kg bw)		
	0	200	600	2000	0	2000	
Glucose (mg/dL)	216.2 ± 12.8	264.1 ± 22.0	261.3 ± 25.2	214.0 ± 17.0	263.2 ± 18.4	245.8 ± 10.2	121.0–197.0
BUN (mg/dL)	18.2 ± 0.9	18.1 ± 0.5	18.8 ± 0.8	19.3 ± 0.8	14.7 ± 1.1	16.7 ± 0.4	10.0–16.0
Creatinine (mg/dL)	0.9 ± 0.0	0.8 ± 0.0	0.9 ± 0.0	0.8 ± 0.0 *	0.8 ± 0.0	0.8 ± 0.0	0.4–0.6
AST (U/L)	121.4 ± 6.0	122.5 ± 8.5	105.8 ± 7.5	124.7 ± 7.3	129.8 ± 11.7	132.8 ± 5.1	77.0–110.0
ALT (U/L)	46.8 ± 1.5	56.6 ± 10.5	46.1 ± 3.7	43.6 ± 2.3	55.0 ± 7.3	55.4 ± 6.5	27.0–46.0
ALP (U/L)	66.3 ± 3.3	68.6 ± 2.5	67.2 ± 3.4	65.1 ± 4.4	65.0 ± 2.5	68.4 ± 6.0	104.0–160.0
Total protein (g/dL)	7.2 ± 0.1	7.2 ± 0.1	7.1 ± 0.2	7.4 ± 0.1	6.8 ± 0.1	7.1 ± 0.2	6.0–7.1
Albumin (g/dL)	3.8 ± 0.0	3.8 ± 0.0	3.8 ± 0.0	3.8 ± 0.0	3.8 ± 0.1	3.8 ± 0.1	3.3–4.6
Globulin (g/dL)	3.4 ± 0.1	3.4 ± 0.1	3.2 ± 0.2	3.7 ± 0.1	3.0 ± 0.1	3.3 ± 0.1 **	-
Total bilirubin (mg/dL)	0.2 ± 0.0	0.1 ± 0.0 *	0.1 ± 0.0	0.1 ± 0.0	0.1 ± 0.0	0.1 ± 0.0	0.2–1.0
Direct bilirubin (mg/dL)	0.1 ± 0.0	0.1 ± 0.0	0.1 ± 0.0	0.1 ± 0.0	0.1 ± 0.0	0.1 ± 0.0	-
Cholesterol (mg/dL)	77.7 ± 4.7	73.9 ± 4.5	70.5 ± 2.8	69.4 ± 5.5	78.8 ± 5.3	82.4 ± 3.9	55.0–89.0
Triglyceride (mg/dL)	187.9 ± 23.4	248.8 ± 26.7	170.9 ± 20.3	135.3 ± 13.5	185.2 ± 25.3	194.6 ± 38.1	62.0–92.0
HDL-C (mg/dL)	51.5 ± 3.3	48.7 ± 3.1	51.7 ± 2.7	50.2 ± 3.4	49.9 ± 2.5	54.9 ± 2.4	-
LDL-C (mg/dL)	10.4 ± 1.2	9.6 ± 0.9	10.1 ± 0.9	10.9 ± 2.1	8.6 ± 1.2	8.8 ± 1.0	-
Uric acid (mg/dL)	5.2 ± 0.4	6.3 ± 0.5	7.1 ± 0.7	6.0 ± 0.4	6.1 ± 0.4	6.5 ± 0.8	-
Na^+^ (mmol/L)	143.4 ± 0.3	143.9 ± 0.4	143.2 ± 0.4	142.8 ± 0.4	144.1 ± 0.7	143.9 ± 1.0	141.0–149.0
K^+^ (mmol/L)	7.1 ± 0.2	8.0 ± 0.4	7.7 ± 0.5	7.0 ± 0.2	7.5 ± 0.4	7.3 ± 0.3	4.6–6.1
Cl^−^ (mmol/L)	98.7 ± 0.6	97.3 ± 0.3	98.7 ± 0.6	100.1 ± 0.3	97.4 ± 0.4	96.9 ± 0.9	101.0–107.0
TCO_2_ (mmol/L)	29.2 ± 0.4	28.8 ± 0.6	29.2 ± 0.5	31.6 ± 0.3 *	30.9 ± 0.9	30.8 ± 0.8	-

Values are expressed as mean ± SEM. *n* = 10 per group (treatment groups), *n* = 5 per group (satellite groups). * Significant difference compared with the control group at *p* < 0.05. ** Significant difference compared with the satellite control group at *p* < 0.05. ^a^ Reference value of 13–22 weeks of Age [23].

## Data Availability

The original contributions presented in the study are included in the article; further inquiries can be directed to the corresponding author.
